# Is It Possible to Prevent Sars-Cov-2 Infection in a Non-Infectious Diseases Ward during the Pandemic on the Example of a Diabetes Clinic Institute of Rural Health, Lublin, Poland?

**DOI:** 10.3390/ijerph18147593

**Published:** 2021-07-16

**Authors:** Piotr Dziemidok, Daria Gorczyca-Siudak, Marzena Danielak

**Affiliations:** 1 Faculty of Human Sciences, University of Economics and Innovation, Projektowa Street 4, 20-209 Lublin, Poland; piotr.dziemidok@op.pl; 2 Diabetology Clinic and Department, Institute of Rural Health in Lublin, Jaczewskiego Street 2, 20-554 Lublin, Poland; mmd24@vp.pl

**Keywords:** SARS-Cov-2, COVID-19 pandemic, diabetes mellitus

## Abstract

Diabetes is considered an epidemic of the 21st century. On 11 March 2020, two months after the outbreak of COVID-19 (coronavirus disease of 2019) epidemic in China, the World Health Organization announced COVID-19 to be a pandemic. From that time, many hospitals and wards have started to function as both infectious and non-infectious ones; so did the Diabetes Clinic Institute of Rural Health in South-Eastern Poland. Considering the global importance of diabetes and its prevalence worldwide, it seemed important to investigate how the Diabetes Clinic passed through the individual phases of the pandemic, and the possibility of protecting hospitalized patients against future pandemic infection. We present detailed characteristics of the situation in a ward which used to treat non-infectious patients with diabetes only and, nowadays, has been obliged to take into account the risk of spreading SARS-Cov-2 (severe acute respiratory syndrome coronavirus-2) infection also. Moreover, we suggest solutions to avoid cases of infectious diseases in non-infectious wards in the future.

## 1. Introduction

### 1.1. Diabetes

Diabetes is the only non-infectious disease to be considered by the United Nations (UN) as an epidemic of the 21st century and belongs to the group of the most important epidemic threats to human life and health [1]. Diabetes is a systemic disease with a multifactorial background. The main clinical and social problems are chronic complications which generate huge social and economic costs [2]. These complications are a real therapeutic challenge. Chronic hyperglycemia leads to damage, functional disorders, and failure of various organs—mainly the eyes, kidneys, cardiovascular system, and nerves. Therefore, diabetes cannot be treated as a single disorder, but as a group of diseases, the common denominator of which is hyperglycemia [3].

In 2019, the number of diabetes-related deaths was estimated at 4.2 million, which is 11.3% of global deaths. The joint expenditures of the health care systems on diabetes are 760 billion dollars—10% of total expenditures. At the forefront of countries with the highest absolute value of expenditure on diabetes are United States (294.6 billion dollars), China (109 billion), and Brazil (52.3 billion). The predictions for total expenditure due to diabetes in 2025, 2030, and 2045 are 438, 578, and 700 billion dollars, respectively, worldwide [4]. In Poland, based on the data by the National Health Fund and the Coalition for Diabetes, there are approximately 3.5 million people ill with diabetes, which means that every eleventh person in the population is ill, including approximately one-third of those who remain undiagnosed [5].

Diabetes and its complications are among the five most frequent causes of death in developed countries. It is recognized that on a global scale each person with diabetes tends to have, on average, a 10 year shorter lifespan [6].

### 1.2. SARS-CoV-2 Coronavirus and COVID-19 Disease

The SARS-CoV-2 (severe acute respiratory syndrome coronavirus-2) is the seventh virus from the family of coronaviruses recognized to infect humans. To this group also belong SARS-CoV, which caused an epidemic during 2002–2003, and MERS-CoV (Middle East respiratory syndrome coronavirus), responsible for an acute infectious disease called Middle East respiratory syndrome, first described in 2012. The remaining coronaviruses from this group are responsible for mild respiratory system infections in humans and animals [7].

COVID-19 is an acute viral disease of the respiratory system caused by SARS-Cov-2 coronavirus. The disease occurred suddenly in the non-immunized population of the Chinese city of Wuhan at the end of 2019 and began to spread in an uncontrolled way. It affected the populations of all countries in the world at lightning speed, triggering a public health crisis on an unprecedented scale.

On 11 March 2020, two months after the outbreak of the epidemic in China, the World Health Organization announced COVID-19 to be a pandemic, with growing numbers of persons infected or deceased daily due to this disease being noted worldwide.

In Poland, the first case was diagnosed on 4 March 2020, and an increase in morbidity resulted in the announcement of the state of pandemic. This was associated with the introduction of a series of restrictions and organizational changes in health care. In many hospitals, planned admissions were cancelled or the functioning of clinics suspended, while parts of hospitals were temporarily converted into single-named infectious diseases hospitals. In the Lublin Province, where the diabetes clinic is located, the first 22 cases were diagnosed on 18 March 2020.

### 1.3. SARS-CoV-2 Coronavirus and Diabetes

The care of patients ill with diabetes or other non-infectious diseases during pandemics is hindered by the fact that the plans of buildings and logistic solutions in internal diseases wards are not suited for mass cases of an infectious disease. In our ward, similar to many others, there is a lack of special sluice rooms with dedicated ventilation, there are no isolation rooms—the rooms being for two three, four, or five persons. Access to the remaining part of the hospital is standard—through lifts and halls. Two other non-surgical wards function in the hospital which are connected by common passageways, but there is a lack of designated routes for infected patients.

During the initial months of the pandemic there was concern that SARS-Cov-2 infection may be more serious in patients with diabetes. This was indicated by preliminary data concerning the situation in Lombardy, Italy. The Polish Diabetes Association takes the view that persons with diabetes are not at greater risk of contracting COVID-19 infection than the general population. However, the problem is the fact that in diabetic patients who developed the COVID-19 disease, a higher risk of severe complications and death was observed, compared to persons without diabetes [8].

Considering the necessity for maintaining the continuity of care of patients with diabetes, especially those who require urgent assistance, despite the pandemic, we made the decision to continue the functioning of the Clinic and treatment of patients for as long as the epidemiological situation allowed. We hoped that taking care of the metabolic control of diabetes and treatment of concomitant diseases would favor better prognosis in the course of potential SARS-Cov-2 infection. Our assessment was later confirmed in writing by the Polish Diabetes Association which stated that a higher risk of a severe course and death occurs only when metabolic control of diabetes is poor [8].

## 2. Materials and Methods

The Diabetes Clinic is a part of the Institute of Rural Health in Lublin, Southern Poland—a hospital comprising of three wards (Diabetes, Internal Diseases, and Rehabilitation Clinics), a multi-specialist outpatient clinic, and adjacent diagnostic facilities. The number of patients admitted to Diabetes Clinic is ~580 annually, i.e., approximately 40–50 per month. The average length of hospitalization in the Diabetes Clinic is 8 days.

In our paper, we analyzed patients admitted to our Diabetes Clinic from March 2020 (the beginning of SARS-CoV-2 epidemic) until March 2021—one year of observation. In order to make our findings clear and suitable for analysis, we divided that time into four phases or stages based on our subjective observations. To our best knowledge our classification has not been used by other centers, but, at least in our opinion, it adequately describes population and social effects of the epidemic. It is important to investigate the possibility of avoiding massive infections of hospitalized patients during epidemics in non-infectious wards. We underline that our Diabetes Clinic has never had additional equipment nor facilities to fight infectious diseases and in a time of the spreading infection special procedures were absent; they were being created, changed, and adapted during the epidemic, adequately to the situation, mainly by heads of the wards. Therefore, it was crucial to make local decisions without waiting for full information or recommendations from the infectious diseases academic centers. Moreover, in many cases we had to develop our own procedures just for our ward because there was no time for waiting or delaying decisions. Because the situation of the Diabetes Clinic was similar to many countries and new waves of COVID-19 infection will, undoubtedly, recur in the future, we consider our experience important to share.

Last but not least, we are not a big scientific center nor infectious diseases center with personnel and procedures dedicated to fight infectious diseases. Like many other non-infectious wards, we were put in a situation which may be described by the words—“just to stay alive, help the patients and keep them protected from the infection”. During the pandemic state, officials and, generally, state resources turn their interest to fight the epidemic, which is perfectly understandable, but on the other hand, many people die from other diseases and their general health situation worsens. It is important to keep settings which deal with patients with non-infectious diseases so that they could be protected from the epidemic and receive proper medical help.

We divided the phases of the functioning of the Diabetes Clinic during SARS-CoV-2 epidemic into the following stages.

Stage 1—during the period March–April 2020: “the first contact with the pandemic, creation of procedures and learning behaviors”.

On 12 March 2020, the authorities of the city of Lublin issued orders concerning the suspension of planned admissions, surgical procedures, and visits in hospitals in the Lublin Province. In order to secure the possibility of hospitalization of patients infected with SARS-CoV-2 virus, the performance of surgical procedures, and provision of other health services, the organization of so-called single-named hospitals was launched dedicated to the treatment of patients with COVID-19. On 8 April 2020, the provincial authorities in Lublin issued a subsequent order concerning triage in non-infectious hospitals, including the Institute of Rural Health. Selection consisted in carrying out regular measurements of body temperature among hospital staff, especially at the moment of entering the hospital area. An obligation was also imposed of the organization of work in the admission room of the hospital emergency unit to enable the safe segregation and isolation of patients with suspicion of COVID-19 from other persons present in the admission room and emergency unit. Zones were selected for patients suspected of COVID-19 without symptoms, and a zone for patients suspected of COVID-19 who presented the symptoms of infection. In the case of implementation of the triage procedure, the hospital was obliged to adjust the principles of segregation and isolation of patients suspected of or ill with COVID-19 to the above-mentioned algorithm of procedure.

From the beginning, that is from March, each patient who reported to the Diabetes Clinic was obliged to wear a mask covering their mouth and nose. In the Admission Room, preliminary triage took place. An interview was carried out concerning the possibility of contact with an infected person or a person suspected of infection, the patient completed an inquiry form, had temperature measured. At the beginning of the epidemic each newly admitted patient had a RT-PCR (reverse transcription polymerase chain reaction) test performed. A change in screening was made over time due to an unacceptable delay in achieving the PCR results. When the delay reached more than 24 h, we decided to use a screening COVID-19 IgG/IgM Rapid Test and in case of a doubtful result the infection was confirmed or excluded by PCR test. The Table 1 presents a procedure of testing which was implemented in the Hospital Admission Room in that time.

New germicidal lamps and air purifiers with HEPA (high efficiency particulate air) filters were purchased for each ward. The decision of buying new lamps was a local one—based on the director’s decision—and had been made before the state of epidemic was officially announced. We used dual-function flow bactericidal and virucidal lamps with an external direct-action radiator which were supposed to provide a full range of disinfecting effects. They gave us the opportunity to provide intensive air disinfection in the presence of people as well as a direct disinfection of the entire room when the staff and patients were outside. UVC (ultraviolet-C) radiation disinfects the air and surfaces (walls, countertops, objects, etc.) reaching various nooks and crannies as reflected rays as well.

By the local decision of the Chief of the Diabetes Clinic, patients’ beds were relocated—the number per room was reduced by one bed, thus increasing the distance between patients to approximately 3 m. Due to the suspension of planned admissions and hospitalizations, the patients had single rooms only in the cases of emergency, or there were a maximum two persons per room unless we had any additional urgent admission. All patients were ordered to wear masks covering the nose and mouth, were forbidden to leave the room, except for necessary examinations, and visits were prohibited. Facemasks, mainly FFP (filtering facepiece)—FFP2 and FFP3, but also surgical masks, for the employees were mandatory according to the in-hospital directive and they were provided by the hospital authorities. The patients hospitalized had they own facemasks brought from home as an obligatory directive, with the possibility to receive a surgical mask on the ward if necessary. The medical staff had to change face masks and surgical coats after every single contact with a patient of unknown status (no PCR evaluation) as well as after every diabetic foot intervention. Working in the outpatient clinic required changing medical clothing. The different kinds of masks (FFP2, FFP3, and surgical masks) were used because of supply shortages especially at the beginning of epidemic. The doctors and nurses had to wear face masks all the time also in the doctors’ and nurses’ rooms.

Previously, meals had been prepared, transported, and distributed to patients in the hall of the Clinic by the staff of the catering company. The Diabetes Clinic had no influence on the way and quality of protective procedures implemented in an outsource provider. Later, from stage 1, it was decided that meals would be divided into portions in disposable containers, collected from a representative of the outsource company at the door of the Clinic, and subsequently distributed to the patients by the staff of the Clinic. Thus, the contact with catering staff was limited and handing over closed portions of meals took place in compliance with safety rules—both sides were obliged to use face masks and disposable gloves.

Stage 2—during the period May–September 2020: “informed prevention”.

After the gradual lifting of restrictions in May, the admission of patients began. From 1 May to 11 October 2020, bed occupancy was 139%, with all recommendations issued in March and April being maintained.

Apart from this, screening PCR tests were introduced for each patient qualified for hospitalization. The day before hospitalization, each patient had to report to the Admission Room to have a nasopharyngeal swab taken. The swab was taken by the fully equipped personnel member (usually a nurse) who wore a FFP2 mask and a full coat dedicated to treat infectious patients. Special hours for taking swabs different from those for admissions or for diabetic foot urgent cases’ consultations were established and executed. Between those hours, the germicidal lamps and air purifiers with HEPA filters were turned on and all rooms and beds were regularly cleaned.

In the case of negative result, on the next day the patient reported to the Clinic. In the case of a positive result, the patient was referred for care by a Primary Health Care physician and reported to the appropriate District Sanitary Inspectorate. Due to the rescinding of the order to work in one facility, the possibility was created for the entire staff of the Clinic to perform the PCR test—to date all results had been negative.

Since the delays in receiving PCR test result ended, we decided to rely on those tests only and that has been the decision to follow till now. The rest of the procedures created in stage 1 were fully implemented. We put the pressure on exact adherence to procedures without exceptions. At this stage, in order to prevent local foci of infection with SARS-CoV-2, anti-epidemic actions at the Institute of Rural Health focused on the strengthening of correct behaviors—maintaining social distance, constant wearing of personal protection means, hand hygiene, and hygiene of rooms and equipment at the Institute. Special attention was paid during changing private clothes into work clothes (including the change of coats while providing medical services in the ward and in the outpatient department), and the separation of the work area from the dining area. The ban on the assembly of several persons in one room at the same time was respected. With respect to patients, the procedure was the same as at the first stage. Additionally, an internal recommendation to avoid eating together in doctors’ or nurses’ rooms was created for the diabetes ward.

During this time, where the general sanitary restrictions were loosened across the country, we tightened the restrictions at the ward. No restrictions in admitting patients to hospital was canceled. All the time, independently of temperature, also during summertime, wearing face masks was required from medical stuff and patients on the corridors and during diagnostic procedures. Only when patients were resting in hospital rooms were they allowed to be without face masks. All the other sanitary rules and restrictions remained. Patients had no family visits, and they were prohibited to go outside of the ward unless for the diagnostics procedures.

Stage 3—during the period October–December 2020: “failure of procedures”.

From 9 November 2020, by a decision of the provincial authorities in Lublin, within the provision of health care in association with prevention, counteracting and control of COVID-19, within the area of infectious and internal diseases the Institute of Rural Health was supposed to select beds for COVID-19 patients. The Institute provided 11 selected beds, including eight observation beds, and one respirator bed for patients with suspicion or confirmed SARS-CoV-2 infection. Due to the lack of the possibility to exclude the whole floor and dedicate it to the treatment of COVID-19, rooms were selected in the Rehabilitation Ward and separated by a temporary sluice.

Internal recommendation by the Chief of the Clinic was made—full separation from other hospital and other wards’ personnel, including auxiliary personnel. Within the ward nurses were asked to drink or eat separately (one person at a time, with two nurses present at the ward). All the executive members and doctors from other units were prohibited to come to the ward without changing protective coats.

Stage 4—during the period January–March 2021: “return to normality in abnormal times”. Generally, there was one change made which concerned serving meals—they were served by the former personnel. Catering representatives were obliged to wear masks and gloves and their behavior was monitored by the ward nurses.

Detailed characteristics of the situation during individual phases is presented in Results.

## 3. Results

### 3.1. Stage 1

During the period from March to April 2020, no infections were noted among the staff and patients of the Diabetes Clinic and the Institute generally. On 30 April 2020, in the area of the Lublin Province a total number of 359 cases of infection with SARS-CoV-2 coronavirus were noted, including 13 decreased and 167 convalescents. From 20 March to 30 April 2020 (i.e., during the period of announcement of the state of pandemic until the stage of lifting the restrictions), 46 patients were hospitalized in the Diabetes Clinic. None of the patients were infected with SARS-CoV-2 coronavirus during hospitalization. Within this period, the Diabetes Outpatient Department at the Institute of Rural Health admitted physically 716 patients and provided 879 on-line consultations.

### 3.2. Stage 2

Starting from 4 May 2020, after approximately 40 days, the loosening of sanitary rules resulted in the doubling of the number of cases in the whole country (from 13,000 to 27,000) and a clear concentration of new foci of infection, including mines in Upper Silesia, at workplaces, and large social meetings, such as first communions or weddings. The pandemic started to spread countrywide, although the balance to date was distorted in the number of the ill and convalescents [9]. Until 30 September 2020 in the Lublin Province, a total number of 2685 infections with SARS-CoV-2 coronavirus was diagnosed, there were 1948 convalescents and 40 deaths, 190 persons were hospitalized due to the suspicion of infection with coronavirus, 6291 were under quarantine, and 150 under epidemiological surveillance.

Discipline of the staff and patients, the limited number of patients, and compliance with the regime produced positive results—during this time, similar to Stage 1, no infections with SARS-CoV-2 coronavirus were noted at the Diabetes Clinic and the Institute generally. 235 patients were hospitalized in the Diabetes Clinic without noting even a single case of SARS-CoV-2 infection.

### 3.3. Stage 3

During the period from October to December 2020, the pandemic situation collapsed throughout Poland, including the Lublin Province, as well as locally at the Institute of Rural Health. On 31 December 2020, 1,294,878 cases of COVID-19 were noted in Poland with 28,554 deaths, and in the Lublin Province, 56,633 cases of infection and 1793 deaths.

The first case of COVID-19 occurred at the Institute in October 2020 when infection by the SARS-CoV-2 coronavirus was transferred from another hospital by a nurse working at the Institute within extra employment. After the thorough investigation we considered this nurse as the “patient zero” of the Institute. At the beginning, she was asymptomatic. During that time, at her primary place of work, there occurred several epidemiologic foci in different wards. Over the course of epidemiological investigation, it turned out that the nurse had contact with an infected person. As a result of importing the virus, infections spread first among patients in the Rehabilitation Ward, and subsequently among nursing staff. In association with the total absence of nursing staff in this ward (persons infected or isolated due to so-called “contact”), the duties were taken over by nurses from other wards at the Institute including nurses from the Diabetology Unit who had been separated from other staff personnel until this time.

After 9 November 2020, i.e., after selecting beds in the Rehabilitation Clinic for patients with suspicion or confirmed SARS-CoV-2 infection, further problems occurred. Even though the selected rooms were placed on another floor, in the following weeks infections were detected among physicians, orderlies, and the remaining staff on different wards at the Institute. During that period, 179 patients were hospitalized at the Institute with the suspicion of COVID-19, and the infection was confirmed in 109 (61%). In that time, we pooled the data from our Diabetes Clinic together with other wards of the hospital because of the spreading of SARS-Cov-2 infection among patients and personnel, resulting in frequent transferring patients between clinics based on their COVID-19 status. Infection was also diagnosed in 39 members of medical staff per 88 working with patients.

In the Diabetes Ward, in the time period between the last week of October 2020 and the first week of November 2020, the only functioning physicians at work were the chief of the clinic and one resident physician. The rest of the doctors were on quarantine since they were positive and/or symptomatic.

The samples for PCR testing were sent to different laboratories depending on their availability. From the beginning the cooperation with labs was satisfactory, but occasionally limited due to the number of samples sent simultaneously from surrounding medical facilities. Fortunately, the number of molecular laboratories increased over time.

### 3.4. Stage 4

We have not had a single case of SARS-Cov-2-positive patient at the ward from the beginning of the COVID-19 vaccination program but we have the same epidemiological procedures even despite the fact that all the personnel were fully vaccinated. We have used masks and performed PCR tests for every patient who was not vaccinated. Patients and hospital staff have had to wear masks all the time, although every day we happen to experience problems with following this rule by the patients. The internal procedure—”if you are not able to wear a mask, you cannot stay at the ward”—has been obligatory. However, patients have been allowed to spend time without masks only in their hospital rooms. From the 1 January 2021 until 7 June 2021, we have admitted 300 patients without even a single case of infection within the ward among patients or personnel. By the end of the period described, we have had three cases of diabetic patients we could not admit due to positive results before the hospitalization started.

## 4. Discussion

In our article, we summarize the results of creating and implementing recommendations in the hospital ward which basically has never been adopted to deal with infectious patients. There are plenty of COVID-19 case reports in the literature but reports from pandemic situations in a non-infectious ward in Poland are in the minority. Such presentations give the opportunity to compare the methods implemented in hospital settings between different types of wards and hospitals.

### 4.1. Stage 1

The time between March and April 2020 was the first period of the pandemic in Poland, during which everyone adapted to the new reality associated with the necessity to function and work during the pandemic. It was a period characterized by fear and uncertainty, among both patients and physicians. Reports from the relevant literature and mass media were changeable and based on single cases. The procedures were still being worked out.

During the pandemic, many hospitals and ambulatories intentionally limited elective visits and admissions to reduce the potential exposure to SARS-CoV-2. Moreover, reductions in urgent or emergency presentations were observed. The consequences of these reductions may contribute to excess mortality associated with COVID-19 pandemic, taking into consideration both infected and not infected patients. As our diabetes ward is a relatively small, we wanted to avoid significant reduction in admissions, especially of patients with the urgent need for hospitalization.

Davison et al., in a scoping review, justified the need for mental health support for various groups of patients with chronic physical diseases during the COVID-19 pandemic. Among others, diabetic and obese patients were included and analyzed. Anxiety and depression were the most common reported mental health conditions for individuals with chronic diseases during COVID-19. Furthermore, several coping practices were described in the reviewed literature but not formally evaluated [10]. From our point of view, the information about the availability of reliable centers treating diabetes and its complications during epidemic may be another factor decreasing the epidemic-related level of anxiety. Nevertheless, in the hospital, i.e., at the Institute of Rural Health (despite the provincial authorities’ recommendations), there was no possibility to organize a second Admission Room for patients with suspicion of COVID-19, so new procedures were developed and implemented, and primarily planned admissions had to be finally discontinued. It was decided to admit patients who required urgent hospitalization—e.g., those with diabetic foot syndrome, at risk of ketoacidosis, decompensated in the course of exacerbation of concomitant diseases, with deteriorated metabolic control of diabetes, and gestational diabetes.

Staff migration was stopped between different places of work and surveillance was intensified over the implementation of procedures concerning the use of personal protection means and hand hygiene. Analysis of the use of personal protection means according to stock availability showed that during this period more than twice as many means were used as in the whole first half of 2019. Each staff member obtained a personal pocket container for a disinfectant, in all rooms—patient rooms, doctor’s offices, nurses’—offices, auxiliary rooms, and passageways, soap and disinfectant dispensers were checked—used dispensers were replaced by new ones, and additional containers were placed at patients’ bedsides. Many decisions, which turned out to be crucial, were made before releasing central recommendations. These were based on the assumption that we live in a global village and there is no such disease as ‘’Wuhan disease” or “Lombardy epidemic” and that we have a global epidemic that will eventually come to us since viruses do not follow arbitrary borders or regulations. During the first months of the epidemic, we implemented personal protective equipment (including surgical, FFP2 and FFP3 masks, face shields, gloves, aprons) and increased hygiene in daily work. Decisions of buying additional UV-lamps and equipment were done without waiting for state recommendations. Those supplies allowed us to survive the time of shortages in medical equipment. In those days till now, increasingly more evidence support the effectiveness of any kind of mask implementation (surgical, FFP2, or FFP3) in medical settings [11]. We also tried to keep distance between workers and patients, but in hospital conditions it is difficult to fully enforce.

After analyzing media reports from January 2020, the medical and management staff felt the need for training on coronavirus-related procedures. The training was obligatory for all the staff members—physicians, including resident doctors, nurses, and orderlies—the characteristics of the virus and preventive measures were presented. Additionally, the chief of the Diabetes Clinic performed daily talks for the personnel such as cleaners and kitchen staff. All those decisions were also made before the central recommendations. Repeatable training and talks as well as reassuring the staff members and patients of being the essential links in spreading the disease were important to maintain compliance. Danish authors showed that introducing collaborative, inclusive and participative practices of staff engagement and involvement instead of traditional organizational cultures that are hierarchical and controlling can be profitable. Our opinion is similar to Hølge-Hazelton et al.: the lack of ward staff experience results in inability to act competently in a crisis like the COVID-19 pandemic, together with the lack of engagement and serious consequences for patients, staff, and the ward managers themselves. [12]. Roma et al. showed the need for strategies using psychological characteristics of people who do and do not comply with the containment measures (i.e., perceived efficacy, risk perception, and civic attitudes) to target their COVID-19 communications more effectively [13].

### 4.2. Stage 2

The subsequent stage of the struggle against COVID-19 began together with the lifting the rigorous restrictions and expanding the functioning of the hospital and Rehabilitation Centre. According to the government plan, the functioning of society and the economy was being restored in 4 stages to the principles from before the pandemic. The necessity to cover the mouth and nose in public spaces remained in place. The period from May to October 2020 was characterized by the loosening of the sanitary rules and issuing of some restrictive recommendations by national institutions.

The biggest task was to force all members of the hospital staff and hospitalized patients to follow procedures introduced in stage 1 although growing optimism and loosening the sanitary restrictions outside the hospital. As an example, the change of procedure for the distribution of meals to the patients was a challenge. Previously, the meals had been prepared, transported, and distributed to patients in the hall of the Clinic by the staff of the catering company. The Diabetes Clinic had no influence on the way and quality of protective procedures implemented in an outsource provider. Therefore, it was decided that meals would be divided into portions in disposable container, collected from the staff of the outsource company at the door of the Clinic, and subsequently distributed to the patients by the staff of the Clinic. Thus, contact with the catering staff was limited, and handling over of closed portions of meals took place with the use of safety principles—both parties were obliged to use masks and disposable gloves.

### 4.3. Stage 3

Hospitals are important settings for diseases’ transmission. Non-infectious wards can be even more dangerous because they are often lacking in procedures dedicated to dealing with infected patients. The outset of the COVID-19 pandemic proved this fact—we could observe the spread of COVID-19 within hospital wards. The literature addressing that subject is still growing. A good example of the devastating risk for health care transmission of SARS-Cov-2 was provided by investigators from St Augustine’s Hospital in Durban, South Africa. The authors documented how a single unsuspected case of SARS-CoV-2 led to six major clusters involving five hospital wards, an outside nursing home and a dialysis unit. The infection was confirmed among 80 staff members and 39 patients, 15 of whom died [14]. A similar situation took place in our ward. Despite adhering to admission rules an unsuspected case of an infected nurse from another ward in the hospital led to a gradual spread of the disease. Moreover, from November 2020, according to an administrative decision a part of one ward at the Institute (i.e., Rehabilitation Clinic) was transformed into observation and corona unit. This solution, however, did not provide full possibility to isolate the infected patients. Medical and auxiliary staff, despite their being separated and using any available means of personal protection, after completing work with suspected or infected patients, stayed in the same rooms with the remaining staff. This created the danger of transmitting the infection onto the subsequent persons. The maintenance of restrictions (e.g., patients moving without vital need), observance of hand hygiene and hygiene of clothes did not produce the expected result at this stage, and the disease spread rapidly. This was associated, among other things, with a high increase in morbidity among the general population, lack of possibility to fully verify contact with infection (false-negative results of the screening tests), and the lack of possibility of hospitalization of patients in single rooms to prevent the spread of infection. Additionally, when, for logistic reasons (lack of staff in other wards), the process began of joining wards of internal diseases profile (in order to provide care for hospitalized patients), the infections intensified. This caused a migration of patients to one ward and common duties of the nurses. These decisions, understandable from the point of view of hospital management and provision of assistance to patients, resulted in a closer contact of nursing staff from various wards and patients in the halls. In addition, auxiliary staff—orderlies worked in one ward which was the combination of two wards, while the managing staff contacted each other and their teams occupying a common space.

After elimination of the foci of infection, the wards returned to normal functioning, although subsequently infections occurred again among the staff, which started from an infection imported from outside. This status lasted until December 2020. Around Christmastime, patients were discharged home in a good state. Staffing was reduced, and the wards were thoroughly washed, decontaminated and disinfected.

### 4.4. Stage 4

On 29 December 2020, the first deliveries of vaccines reached Poland. The National Programme of Vaccinations Against Sars-Cov-2 was developed, which assumed that at first there would be no mass vaccinations, and the whole action will be divided into stages:

Group 0: medical staff, staff of nursing homes, administration workers in medical facilities and sanitary stations.

Group 1: residents of nursing homes, uniformed services, persons aged over 60 with indications to first provide the oldest people, those occupationally active, and with concomitant diseases.

Group 2: teachers, public transport workers, officers directly engaged in control of the pandemic, persons aged under 60 with chronic diseases increasing the risk of a severe course of COVID-19.

Group 3: entrepreneurs from the sectors closed due to restrictions.

### 4.5. Summary of Actions Undertaken in the Diabetes Clinic

After analysis of media reports from January 2020, there was a need for training on coronavirus-related procedures. The training was obligatory for all the staff members in which physicians, resident doctors, nurses, and orderlies participated and the characteristics of the virus and preventive measures were presented. Additionally, the chief of the Diabetes Clinic performed additional daily talks for the additional personnel, e.g., cleaners and kitchen staff. Special attention was paid to the importance of washing and disinfection of hands to prevent the spread of infection. Each member of staff received a pocket container of disinfectant, with possibility of its cyclic refilling. In addition, containers with hand sanitizer were placed, apart from standard sites, at entrances and in corridors and passageways. Patients were also instructed about the necessity for hand hygiene. Information posters were hung in many places—with diagrams of hand washing and disinfection, algorithms of procedure in the case of suspicion of infection, and management of waste and infectious material.

The obligation to maintain social distance was introduced, as well as the constant wearing of protective masks, and use of other personal protection means according to the current needs. The staff received shield masks, face masks, gloves, disposable kits, googles and overalls were available without limitations. A ban was introduced on wearing the same clothes in the Clinic and Outpatient Department; additional clothes was purchased for each staff member. In addition, the staff limited their work only to Diabetes Clinic in the Institute at stage 1 which decision was impossible to maintain during later stages due to economic and legal issues. Patients hospitalized in the Clinic were obliged to wear masks, especially during doctor’s visits, diagnostic tests, moving to the laboratory or for dressings. With the exception of necessary situations (procedures, examinations) the patients were forbidden to move around the area of the Clinic and the Institute. When the use of rehabilitation procedures was necessary, the patients had specified hours in order to prevent contact with patients from outside the Institute and from other Clinics. During a doctor’s visits and during procedures in the hall during rehabilitation, the patients were obliged to wear masks. During this time, we were admitting the most severe cases of diabetic foot due to previous and still present limitations in the availability of professional help in hospitals. Many of those people needed rehabilitation to restore the ability to walk or even seat. Meals prepared by the outsource company were packed in disposable containers, after collection from the lift on the level of the Clinic by the nursing staff of the Diabetes Clinic, they were passed to the patients in hospital rooms.

In 2019, the podiatry consultation room was transferred from the Diabetes Clinic to the Admission Room, a separation which happened to be of key importance in the pandemic situation—there was no contact of patients between Clinics. In addition, some patients who reported to the Admission Room with diabetic foot syndrome were consulted prior to admission to the Clinic, and subsequently the decision was made concerning hospitalization or its postponement, of referring the patient to another facility.

During the period of increased exposure, special attention was paid to the maintenance of general and microbiological cleanness of the rooms in the Clinic, especially frequently touched surfaces and items. The orderlies were trained in the principles of the order for cleaning and the selection of cleaning and disinfecting agents. The use of disinfectant wipes was recommended instead of agents in the form of sprays, in order to limit the splatter of aerosol. Irradiation with UV lamps and air ionization with purifiers—ionizers with HEPA filters—were implemented.

Our own experiences show that the prevention of infections with the SARS-CoV-2 coronavirus is possible. Until 11 October 2020, no cases of infection were noted in the Diabetes Clinic, neither among the patients nor the staff. The implementation of clear procedures and rules of conduct, the same for everyone, and strict adherence to them produced the expected results. The use of screening tests helped to detect infected persons prior to admission to the Clinic, and consequently, eliminate the source of infection. Loosening of the restrictions, however, led to cases of infection among the staff and patients.

The reasons behind the difference between our hospital that had not been treating infected patients at the beginning of the SARS-Cov2 pandemic and other hospitals that had are unknown. Such evaluation should be done, but rather from the perspective of health authorities than ours, as we do not have the resources nor the authority to do so. From our point of view there are two main differences—the size of the hospital (in our hospital the number of potential places where patients from different wards could contact is limited) and the scale of the medical staff migration between hospital departments (in our hospital relatively small). The problem occurred when the staff migrations increased in phase 3.

The last question is whether we were lucky or brought in the right procedures at stage 1, stage 2, and stage 4. We think both reasons are correct. At the beginning, in stage 1 we were learning how to cope with the new reality and every day we were facing new problems without developed procedures. Possibly, in case the infection rate in Lublin Province was higher we could not have managed to have 100% non-infected patients. From the step 2 until now, it probably has been accomplished most thanks to our own procedures and involvement in making the Diabetology Clinic a safe place. We paid special attention to making auxiliary personnel feel important. We also assumed that when you deal with tired people (both patient and personnel), quite often scared, there is an urgent need to repeat daily duties and explain a proper way of doing small things—using face masks, UV lamps, cleaning the beds after every patient, responding to patients’ behavior. When the chief of the ward, doctors, nurses lose control of basic elements—everything can collapse. During an epidemic it seems to better assume that if something may go wrong it will go wrong. Last but not least, every member of the hospital staff has to control himself and must be allowed to make suggestions for improvements, because the management staff is not infallible. Obviously, during pandemic both patients and professional staff were under high emotional distress what probably can explain why many of the patients at stages 2 till 4 had troubles with adhering to the rules. Nevertheless, all involved groups should be informed what are the red lines and what kind of behaviors cannot be accepted.

We are strongly convinced that official recommendations from the government and infectious diseases centers are released often too late therefore it is always crucial to be up to date with the newest peer reviewed research on the subject and the local epidemiological situation in order to respond adequately and timely. Clinical intervention while waiting for the recommendations could prove counterproductive and result in an inability to contain the spread of COVID-19 inside a hospital.

Future measures: In order to avoid cases of other infectious diseases in the future, attention should be paid to the so-called “mobile staff”—it is our working name for persons migrating from one ward to another. This includes auxiliary staff—orderlies, cleaners, kitchen workers, and hospital managers—board of directors, heads of the wards, and head nurses. In the case of detection of infection in the ward, it should also be isolated from the aspect of staff flow. It would be advisable to create permanent teams of nurses, physicians, and auxiliary staff, with the separation of separate employee cloakrooms in order to minimize contacts. In a case of having even a single case of infected patient on the ward, all patients should be discharged with subsequent quarantine in home conditions, or hospitalization in the Infectious Diseases Ward. The non-infectious ward—in this case the diabetes clinic—should be decontaminated and disinfected, and opened not earlier than after the isolation period for a given pathogen. In the case of COVID-19, swabs should be taken from the staff 7–10 days after the last infection in the ward, or other confirmed contact with infection. Viral infections, by their nature, are more difficult to control than bacterial infections, if only because the size of the viral particles, or increased possibility of transformation into sub-strains, or even mutations.

It seems that establishing infectious beds in non-infectious wards should be avoided in the future because there is an increased risk of transmission of viral particles between the ill and healthy patients, and the risk of infection of the staff providing medical care (physicians, nurses), auxiliary staff (orderlies, kitchen orderlies, personnel distributing meals), as well as management staff (charge nurses, head nurses, heads of the wards, directors and other management staff). In practice, a total ban should be introduced on in-hospital migration, starting from the moment of entering hospital to the moment of leaving the premises. The functioning of air condition, which should not be common for all wards, is also an issue for consideration. In the case of the lack of possibility to isolate air conditioning systems, total turning off should be considered for the time of epidemic.

A much better method is the creation of so-called single-named temporary hospitals, which can be established even in stadiums, where the possibility of migration of the virus from carriers is hindered, and from the beginning to the end, the staff is focused and aware of the risk.

The subsequent weak points are the systems for moving around, and the location of “COVID” wards on high floors seems to be a wrong solution. An exception are solutions which allow the separation of lifts and other passageways from those for patients burdened with other health problems, and the staff dedicated to providing services for these patients.

From the point of view of the treatment of the community there are three groups of patients:

1. Patients with an infectious disease.
2. Patients with other health problems.
3. Patients with other diseases who, at the same time, are carriers of an infectious disease.

Points 1 and 2 are relatively obvious and should be treated by an entirely different staff in separate places, group 3—let us give it a working name “a mixed group”, which is burdened with an infectious disease or other disorder requiring intervention, may evoke doubts. Taking into account our experiences, this group of patients should be provided care in single-name hospitals of the highest referential level. Such a hospital should have within reach the performance of computed tomography (CT) and magnetic resonance (MR), vascular examinations, an internal diseases ward, surgical ward, and specialist wards: neurological, neurosurgical, surgical, interventional cardiology, and rehabilitation. It seems that if such care is not sufficient, the patient may be transferred to a specialist hospital not dedicated to the treatment of an infectious disease, assuming the highest level of safety principles.

It should be emphasized that the procedures worked out globally during the COVID-19 pandemic occurred to be ineffective—in the case of a more dangerous pandemic, burdened with higher morbidity, a longer period of asymptomatic course, or another coronavirus associated with a more severe course, these procedures would not protect the staff against infection, or could even be the cause of intensification of the pandemic. Pandemics more dangerous than SARS-CoV-2 cannot be excluded; therefore, recommendations should be implemented not only of a global, but also a national character, which would consider local specificity and even social habits and customs in a given area. Without the implementation of such measures, at least outline solutions and plans, the pandemic and epidemiological future of the world should be seen in dark colors. In addition, the inertia which requires analysis of only randomized studies, does not work in the case of a developing epidemic. Such conduct may lead to the situation in which the global and Polish epidemiological system would be ready for struggle with the previous pathogen, but not to confront a new threat.

Our perspective, qualitative paper has several limitations. The study design is neither a randomized nor an observational as the described interventions were the responses to changing conditions during COVID-19 pandemic. Our work is anecdotal, but we aimed to create it so that it consists of gathered information about how a non-infectious ward may cope with a new infectious disease. Furthermore, materials and methods were changing but the dynamic situation required it. Our results may not capture perspectives of physicians practicing in other parts of the world or in other specialties. Furthermore, the dynamic nature of the pandemic makes it probable that new challenges not identified in our paper will arise over time. Despite these limitations, we believe that our findings will be helpful in comparing methods used to prevent SARS-Cov-2 spread within small internal medicine wards and hospitals.

## 5. Conclusions

The avoidance of COVID-19 infections in our Diabetology Clinic during 3 out of 4 stages of the epidemic was rather connected to flexible decision making and consequence in executing actual recommendations.

In a time of epidemic only teamwork counts—the ward is as good as the most modest member of the team is.

Everyone makes mistakes. If the auxiliary personnel are not allowed to pay attention to the mistakes of the members of executive personnel, the latter get out of control.

Time is one of the biggest enemies. As the time of the epidemic passes people are getting tired of the standards and regulations. Especially for the internal personnel, daily reminding of the reasons for such regulations is mandatory.

The inertia and fear of making mistakes is another enemy. You cannot wait for the central recommendation at the beginning of an epidemic—because it is obvious that they will come too late. It is better to do something instead of not doing anything.

If you wait for type A recommendations during the beginning of a pandemic, you will always be too late. If you do not know what to do, use common sense.

Ask auxiliary personnel what can be done differently. They know the weak points of every day routine better than you do.

If you created the rules—then they are for everybody—also for the director, board members, visiting prime minister—as for cleaning personnel. The viruses are not very good at recognizing charges and scientific titles. From the epidemic point of view the auxiliary personnel’s dedication to follow the rules is equally important as specialists’.

Table 2 and Table 3 present in-hospital risk factors of the spread of SARS-Cov-2 infection, which are modifiable and difficult to modify, respectively (based on experiences of the Diabetes Clinic at the Institute of Rural Health in Lublin).

## Figures and Tables

**Table 1:** Testing procedures during the admission to the hospital.

The COVID-19 IgG/IgM Rapid Test Result	Epidemiological Status	Procedure
negative	negative	admission to the ward
positive or questionable	-	PCR testing, waiting for the result in the isolation room of the admission room (until the result was obtained, the patient was considered potentially infectious)
-	positive or questionable	sending the patient home and placing under the supervision of primary health care or sending to single-named infectious diseases hospitals in case of more serious condition

**Table 2:** Modifiable risk factors of spread of SARS-Cov-2 infection.

Migration of the staff—auxiliary and management between wards and hospitals. Common consumption of meals by medical and nursing staff. Inappropriate selection of hospitals and wards for conversion into hospitals dedicated to treatment of infectious and non-infectious diseases at the same time.

**Table 3:** Risk factors for spread of SARS-Cov-2 infection difficult to modify.

False-negative tests results. Contact of working staff outside the place of work—family, shopping, commuting to place of work. In-hospital air conditioning or its lack, lack of proper technical surveillance. Interpersonal contact during long period of staying together—8, 12, 24 h working in one room and/or when on duty. Interpersonal contact in the case of the lack of isolated lifts and passageways. Tiredness of personnel and patients as time of epidemy is prolonging.
